# Time-Optimized High-Resolution Readout-Segmented Diffusion Tensor Imaging

**DOI:** 10.1371/journal.pone.0074156

**Published:** 2013-09-03

**Authors:** Gernot Reishofer, Karl Koschutnig, Christian Langkammer, David Porter, Margit Jehna, Christian Enzinger, Stephen Keeling, Franz Ebner

**Affiliations:** 1 Medical University of Graz, Department of Radiology, MR-Physics, Graz, Austria; 2 University of Graz, Department of Psychology, Graz, Austria; 3 Medical University of Graz, Department of Neurology, Graz, Austria; 4 Siemens AG, Healthcare Sector, MR R&D, Erlangen, Germany; 5 Medical University of Graz, Department of Radiology, Division of Neuroradiology, Graz, Austria; 6 University of Graz, Institute for Mathematics and Scientific Computing, Graz, Austria; University of Alberta, Canada

## Abstract

Readout-segmented echo planar imaging with 2D navigator-based reacquisition is an uprising technique enabling the sampling of high-resolution diffusion images with reduced susceptibility artifacts. However, low signal from the small voxels and long scan times hamper the clinical applicability. Therefore, we introduce a regularization algorithm based on total variation that is applied directly on the entire diffusion tensor. The spatially varying regularization parameter is determined automatically dependent on spatial variations in signal-to-noise ratio thus, avoiding over- or under-regularization. Information about the noise distribution in the diffusion tensor is extracted from the diffusion weighted images by means of complex independent component analysis. Moreover, the combination of those features enables processing of the diffusion data absolutely user independent. Tractography from in vivo data and from a software phantom demonstrate the advantage of the spatially varying regularization compared to un-regularized data with respect to parameters relevant for fiber-tracking such as Mean Fiber Length, Track Count, Volume and Voxel Count. Specifically, for in vivo data findings suggest that tractography results from the regularized diffusion tensor based on one measurement (16 min) generates results comparable to the un-regularized data with three averages (48 min). This significant reduction in scan time renders high resolution (1×1×2.5 mm^3^) diffusion tensor imaging of the entire brain applicable in a clinical context.

## Introduction

Diffusion weighted imaging (DWI) has become one of the most important methods in magnetic resonance imaging (MRI) to study structural characteristics of cerebral white matter (WM) [1]. DWI provides the basis for diffusion tensor imaging (DTI) that enables the derivation of various parameters such as fractional anisotropy (FA), trace or apparent diffusion coefficient mapping which have been shown to have a high clinical impact in stroke [2], Alzheimer’s disease [3] and multiple sclerosis [4]. Furthermore different methods have been developed to visualize the orientation of fiber bundles such as deterministic tractography (DT) [5], [6] and probabilistic tractography (PT) [7]–[9]. The widespread applications of these techniques to the field of neuroscience include fundamental psychological studies about individual differences of white matter architecture [10], structural connectivity [11], investigation of structural white matter changes in neurodegenerative diseases [12]–[14], pre-surgical mapping [15] and much more.

The most widely utilized method to acquire DWI data is based on diffusion weighted single-shot echo planar imaging (ss-EPI) MR pulse sequences. These sequences are fast but limited in spatial resolution due to the need of acquiring the entire k-space in a single readout shot. To overcome this limitation, there have been a number of developments in MR pulse design that enable the acquisition of high-resolution, diffusion-weighted images with reduced susceptibility artifact and low sensitivity to motion-induced phase error. One such development is 2D navigator-corrected, readout-segmented EPI (rs-EPI) with 2D-navigator-based reacquisition [16], [17]. This technique improved the original rs-EPI method [18] by adding 2D non-linear phase correction [19] and navigator-based reacquisition, based on previous work with 1D navigators [20]. The technique achieves a low level of susceptibility artifact by allowing a very short echo spacing in the EPI echo train and the artifact is further reduced by combining the technique with parallel imaging using GRAPPA [21]. Despite the undisputed merits of this novel technique, [22]–[26], providing DWI data with a possible resolution below one millimeter, the applicability of this method suffers from long scan times necessary for acceptable signal to noise ratio (SNR).

One common approach to improving SNR in medical imaging is denoising the data by means of regularization. Many attempts have been made to regularize ss-EPI data based on the regularization of the DWIs [27], regularization of the diffusion tensor [28]–[31] or spectral regularization of the tensor’s eigenvalues and eigenvectors [32]. Given the fact, that scan time reduction is not a crucial task in ss-EPI DWI, the focus of this technique has been the improvement of image homogeneity.

However, one of the most challenging tasks in medical image denoising is to avoid smoothing and hence suppressing essential anatomical details. Several approaches based on partial differential equations (PDE) have been proposed to regularize noisy data whilst preserving discontinuities. One of the most prominent methods to address this problem is the total variation method (TV) introduced by Rudin, Osher and Fatemi usually referred to as the ROF model [33]. TV regularization based on the assumption of piece-wise constant signals has successfully shown to have an outstanding ability to denoise images whilst preserving edges. In recent years, a lots of research efforts has been put into study and further development of the ROF model [34]–[36] and apply TV regularization to scalar images, vector images [37] such as color images and even tensor valued images, including diffusion tensor images [28]–[30], [38]. An algorithm that has raised attention in image restoration because of its simplicity, robustness and calculating speed has been proposed by Chambolle et al. [39]. We took advantage of this algorithm that was originally proposed for scalar valued data and extended this specific method to tensor valued data in order to regularize the entire diffusion tensor.

The regularization parameter that determines the amount of regularization is usually constant assuming the same noise level over the entire image. This assumption is not valid in MRI given that the SNR depends on the distance to the coil surface due to its coil sensitivity [40] and the noise level across the image is modulated by physiology, g-factor in parallel imaging and normalization procedures. Considering the fact that the entire diffusion tensor was regularized in our proposed method, a corresponding noise tensor was evaluated for spatially dependent regularization. The information about the spatial noise distribution for each diffusion tensor element was generated by extracting the noise from DWI data and projecting the noise into the diffusion tensor subspace. Once the spatial noise information for the diffusion tensor was available, an automatic adaption of the spatially varying regularization parameter was possible because the noise level, incorporated in the cost-function to be minimized, determined the stopping criteria. The noise extraction from DWI data was realized using independent component analysis (ICA), a multivariate method to separate different signal contributions according to their statistical properties.

Originally, ICA was proposed to solve the blind source separation problem (BSS) that aims to decompose a mixed signal into several statistically independent components and belongs to the wide class of unsupervised learning algorithms. The basic linear ICA model supposes that a measured signal is a weighted linear sum of underlying independent components (ICs). These signal components can be separated without a priori knowledge about the sources by maximizing the statistical independence for the estimated components. Several algorithms with different theoretical bases have been developed to solve the BSS problem by means of ICA [41], [42]. Common approaches are based on higher-order statistics for measuring the signal’s non-Gaussianity and second-order statistics for exploring sources with temporal structures or non-stationary properties. However, in MRI ICA has been applied for separating activation patterns from fMRI measurements [43], correction of vascular signal contribution in dynamic susceptibility MRI (DSC-MRI) [44]–[46], enhancing the contrast of gray and white matter [47], assessment of cerebral blood perfusion from dynamic contrast enhanced MRI (DCE-MRI) [48], application to diffusion tensor imaging [49] and to other fields of MRI [50]–[52].

In this work we show that the proposed regularization significantly improves tractography of high-resolution DWI data with low SNR due to short acquisition time. The novel regularization algorithm applied to the diffusion tensor has two attractive features. Firstly, an automatic calculation of the regularization parameter was performed by using the noise information from DWI data making our approach user independent. Secondly, given the fact that the SNR is spatially nonuniform distributed due to the spatial dependence of the coil sensitivity, the regularization was forced to vary spatially. The noise distribution required for this procedure was extracted from DWI data by means of complex independent component analysis. Tractography was performed for a software phantom and for high resolution in vivo data to study the performance of our proposed regularization algorithm. Tractography relevant parameters such as Mean Length (ML), Track Count (TC), Volume (V), and Voxel Count (VC) were evaluated and compared for DWI measurements from one average, two averages, three averages, and for one average with applied regularization respectively.

## Materials and Methods

### 1.1 MRI-Measurements

DWI data from two healthy volunteers were acquired using a rs-EPI sequence with the following parameters: TR = 5600 ms, TE = 70 ms, FOV = 240 mm, resolution = 1×1×2.5 mm^3^, slices = 38, b = 1000 s/mm^2^, diffusion directions = 12, number of shots (or readout segments) = 11, EPI echo spacing = 0.34 ms, acquisition time = 48 min, averages = 3, GRAPPA acceleration factor R = 3. For further processing, data sets from one, two and three averages were evaluated separately. The regularization of the diffusion tensor was applied only on the data from the first average (acquisition time = 16 min). All measurements were carried out on a MAGNETOM 3 T Tim Trio system (Siemens AG, Healthcare Sector, Erlangen, Germany) using a 32 channel head coil. The volunteers gave written informed consent and the study was approved by the local ethics committee of the Medical University of Graz.

### 1.2 Preprocessing

To correct for geometrical distortions eddy current correction was performed using FMRIB’s Diffusion Toolbox (FDT v2.0) a part of the FMRIB Software Library (FSL v4.1). Afterwards, segmentation of the brain was carried out using the Brain Extraction Tool (BET v2.1) that is also included in FSL.

### 1.3 Independent Component Analysis

**Figure 1:**
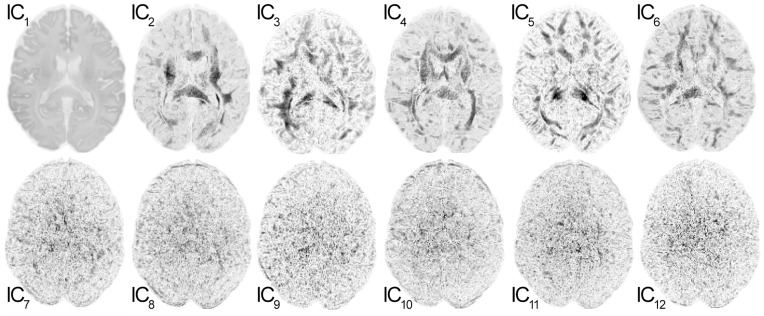
Twelve independent components obtained by means of ICA from 12 The components IC_1_–IC_6_ (upper row) contain information about tissue microstructure and directed diffusion, the components IC_7_–IC_12_ (lower row) contain noise information. Images are scaled equally and displayed in inverted view.

ICA was performed on each slice of DWI data for all 12 directions with diffusion sensitizing gradients (b = 1000 s/mm2), based on the data from the first out of three averages. The algorithm used in this work, referred to as “complex ICA by entropy bound minimization” (complex ICA-EBM), is based on the principle of maximum entropy and applies a line search optimization procedure using a projected conjugate gradient [53]. An implementation of this algorithm in MATLAB software (The Mathworks, Inc., Natick, MA), is available at: http://mlsp.umbc.edu/ica_ebm.html. As a result of this transformation a series of 12 independent components was obtained (IC1, IC2, …, IC12) in which the first six components contain information about tissue and diffusion in six independent directions and six components contain noise (see figure 1). By setting the noise components (IC7–IC12) to zero and inverting the ICA transformation, a denoised DWI series was obtained, denoted as/with i = 1,…, N the number of diffusion encoding directions. In a second step, the ICs containing information about tissue and diffusion (IC1–IC6) were set to zero prior to the ICA back transformation, to achieve a DWI corresponding noise series denoted as 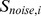 again, with i = 1,…, N the number of diffusion encoding directions. Both, the denoised DWI data and the corresponding noise data were used to evaluate the diffusion tensor and a corresponding noise tensor.

### 1.4 Evaluation of the Diffusion Tensor and the Noise Tensor

The signal intensity *S* in DWI and the diffusion tensor **D** are related through the Stejskal-Tanner equation [54], [55] as given by
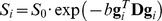
(1)where *S_0_* is the signal without diffusion gradient (b = 0 s/mm^2^), **g** is the diffusion encoding unit vector and *b* the diffusion weighting. The superscript *T* denotes the vector transpose and *i = 1,…, N* the number of diffusion encoding directions respectively. We followed the most widely used method to estimate the diffusion tensor, solving the linear least square (LLS) problem by minimizing the objective function:



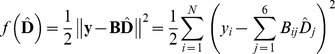
(2)where



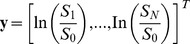
(3)accounts for the linearization of Eq. [1],



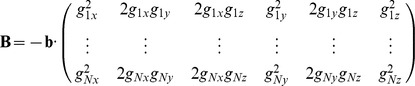
(4)is the encoding gradient design matrix and



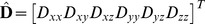
(5)is a vector representation of the diffusion tensor. The solution to the LLS problem is given by:



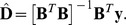
(6)


With this, the diffusion tensor 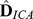 was evaluated from the denoised DWI data 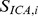 that served as input for Eq. [3].
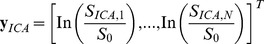
(7)


From the DWI corresponding noise series 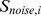 a noise tensor 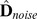 was evaluated using Eq. [6] with a modification of Eq. [3] given by
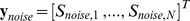
(8)


Given the fact that the noise was projected into the same subspace as the diffusion tensor, 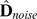 provides spatial information about the noise distribution in 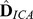. This spatial information was incorporated into the regularization process in order to apply a spatially varying regularization of 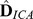.

### 1.5 Spatial Total Variation Regularization

The ROF model introduced by Rudin et al. [33] aims to minimize the Total Variation of *u* and is defined by the following variational model:
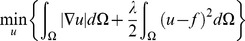
(9)where Ω is the image domain, *f* is the noisy image, *u* is the sought solution and the parameter λ controls the strength of regularization. A simple and efficient algorithm that solves the dual formulation of the ROF model [34] has been proposed by [39]. Furthermore, an automatic update of the regularization parameter λ is suggested if the noise, given by the variance σ, is known. In every iteration step the adaption of λ follows the rule:



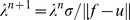
(10)


A generalization of the Total Variation seminorm for scalar-valued data 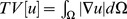 was given by [37] for vector valued data and was extended to matrix valued data by [30]. The Total Variation norm of a matrix 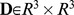 is defined as:
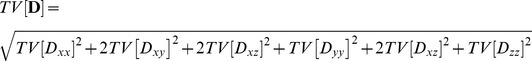
(11)


For the spatial regularization of the diffusion tensor, the regularization parameter 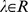 was replaced by a regularization tensor 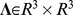. With this and Eq. [9] the modified objective function to be minimized is given by:
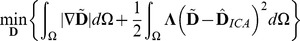
(12)where 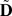 is the sought regularized diffusion tensor and 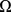 the tensor space. The noise distribution of the diffusion tensor is given by the estimated noise tensor 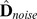. For the automatic update of the regularization tensor a noise variance tensor 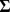 was estimated by averaging the variance in a 5×5 pixel moving window for all elements of 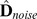. This transforms Eq. [10] to the tensorial update rule:



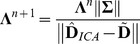
(13)


**Figure 2:**
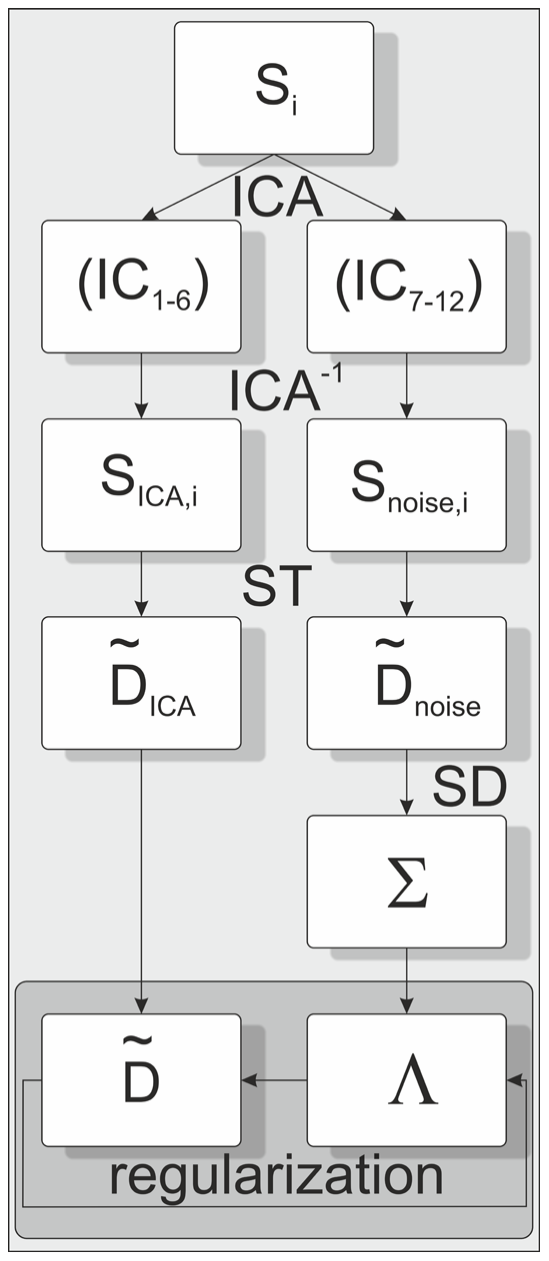
Schematic overview of the adaptive spatially varying regularization algorithm. 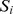
 denotes the diffusion-weighted images, IC the independent components decomposed by ICA, 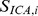 are the denoised diffusion-weighted images with the corresponding noise images 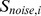 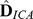 is the diffusion tensor with the corresponding noise tensor 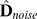, evaluated using the Stejskal-Tanner equation (ST). The noise variance tensor 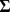 was estimated by averaging the variance in a 5×5 pixel moving window (SD). 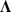 denotes the regularization tensor to estimate the sought regularized diffusion tensor 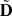.

The initialization of 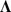 is given by 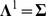. All calculations were performed using MATLAB software (The Mathworks, Inc., Natick, MA). A schematic overview of the algorithm is given in figure 2.

### 1.6 Software Phantom

In order to create a noise-free gold standard for tractography, a software phantom was constructed corresponding to the dimension of the in vivo data using MATLAB software (The Mathworks, Inc., Natick, MA). This software phantom constructed in the tensor subspace included fibers in all three spatial directions. Subsequently noise from ten different measurements was added to the phantom to study regularization properties. To ensure that the added noise is comparable to noise from in vivo data, DWI measurements were performed on a homogenous water phantom in order to obtain the noise information by means of ICA. Scan parameters were kept constant for the water phantom and for in vivo measurements. The noise tensor was evaluated and added to the software phantom.

### 1.7 Tractography

For both, the software phantom and in vivo data, tractography was carried out using Diffusion Toolkit (Ruopeng Wang, Van J. Wedeen, Athinoula A., Martinos Center for Biomedical Imaging, Massachusetts General Hospital, Boston, MA) in which the regularized and native tensors served as input data. Fiber tracts were visualized using TrackVis software (Ruopeng Wang, Van J. Wedeen, Athinoula A., Martinos Center for Biomedical Imaging, Massachusetts General Hospital, Boston, MA). For the software phantom the (i) noise free data, (ii) the noisy data and (iii) the noisy data with regularization have been analyzed with respect to the parameters Mean Length, Track Count, Volume and Voxel Count obtained from TrackVis Software. The same parameters were evaluated for in vivo data from two subjects for the entire brain. For assessing tractography parameters from measurements acquired within 16 min (rs-EPI_1_) all three data sets were evaluated separately (1, 2, 3) and parameters were averaged afterwards. Similarly, for measurements acquired within 32 min (rs-EPI_2_) three combinations of two averaged data sets were used (1+2, 2+3, 1+3). For assessing tractography parameters from measurements obtained within 48 min (rs-EPI_3_), all three data sets were averaged (1+2+3). The evaluation of parameters from regularized data (rs-EPI_1,reg_) based on rs-EPI_1_ was again performed separately. The angle threshold was fixed to 45° for the tractography analysis for all data.

## Results

**Figure 3:**
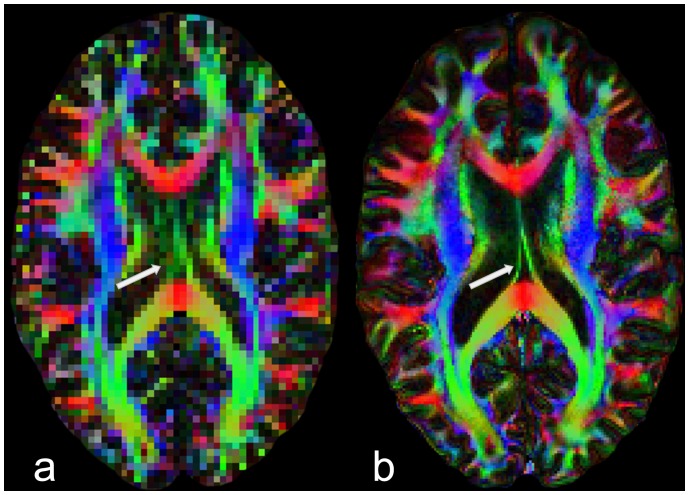
Comparison of FA maps from conventional ss-EPI data (a) with a resolution of 2.5×2.5×2.5 mm^3^ with high resolution rs-EPI data (b) with a resolution of 1×1×2.5 mm^3^. Small structures such as the fornix (marked by the arrow) or branches in peripheral regions can hardly be seen in conventional DTI scans with limited resolution but can be clearly identified in high resolution rs-EPI.

Exemplarily, the gain of spatial resolution in rs-EPI compared to standard ss-EPI scan with a resolution of 2.5×2.5×2.5 mm^3^ is demonstrated in figure 3. Small structures such as the fornix which can hardly be visualized in the conventional low resolution scan can be accessed by high resolution rs-EPI.

**Figure 4:**
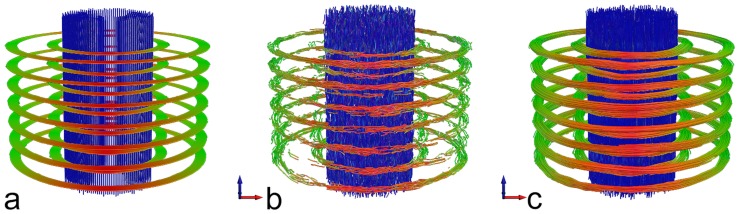
Tractography from the software phantom without noise (a), from the phantom with overlaid noise (b) and from the noisy, regularized phantom (c) demonstrating the improvement of fiber homogeneity due to regularization.

Visual inspection of the tractography for the noise-free software phantom, the noisy phantom and the regularized noisy phantom showed that the proposed processing of the diffusion tensor improved the homogeneity of fiber orientation (see figure 4). This observation was quantitatively confirmed when evaluating the tractography parameters Mean Length, Track Count, Volume and Voxel Count (table 1) revealing that values for the noisy regularized phantom are close to values obtained from the noise free software phantom.

**Table 1:** Evaluated tractography parameters Mean Length (ML), Track Count (TC), Volume (V), and Voxel Count (VC) for the noise-free phantom, the phantom with overlaid noise from ten measurements (mean ± standard deviation) and the noisy phantom with regularization (mean ± standard deviation).

	phantom without noise	phantom with noise	phantom with regularization
**ML (mm)**	135.5	22.2±1.7	132.5±7.8
**TC (1)**	55596	51743±722	55701±368
**V (ml)**	179.0	172.9±2.1	179.1±1.0
**VC (1)**	57290	55522±145	57368±112

**Figure 5:**
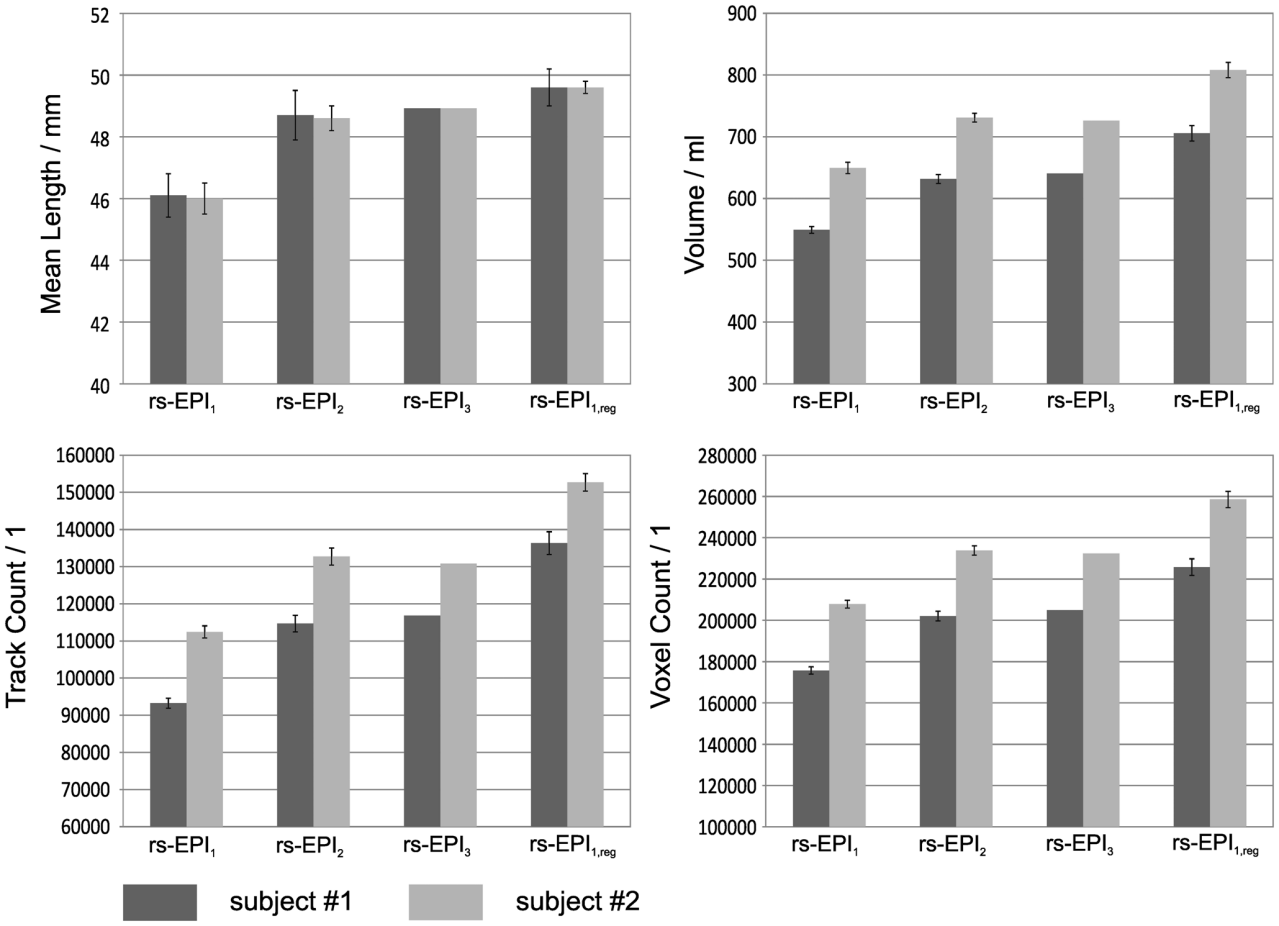
Evaluation of tractography parameters Mean Length, Track Count, Volume and Voxel Count for rs-EPI data obtained from one average (rs-EPI_1_), two averages (rs-EPI_2_), three averages (rs-EPI_3_) and from one average processed with the regularization algorithm (rs-EPI_1,reg_) for the entire brain for both subjects. Please note that errorbars in rs-EPI_1,_ rs-EPI_2_ and rs-EPI_1,reg_ denote the standard deviation due to the separate evaluation of all three measurements and their combination respectively.

**Figure 6:**
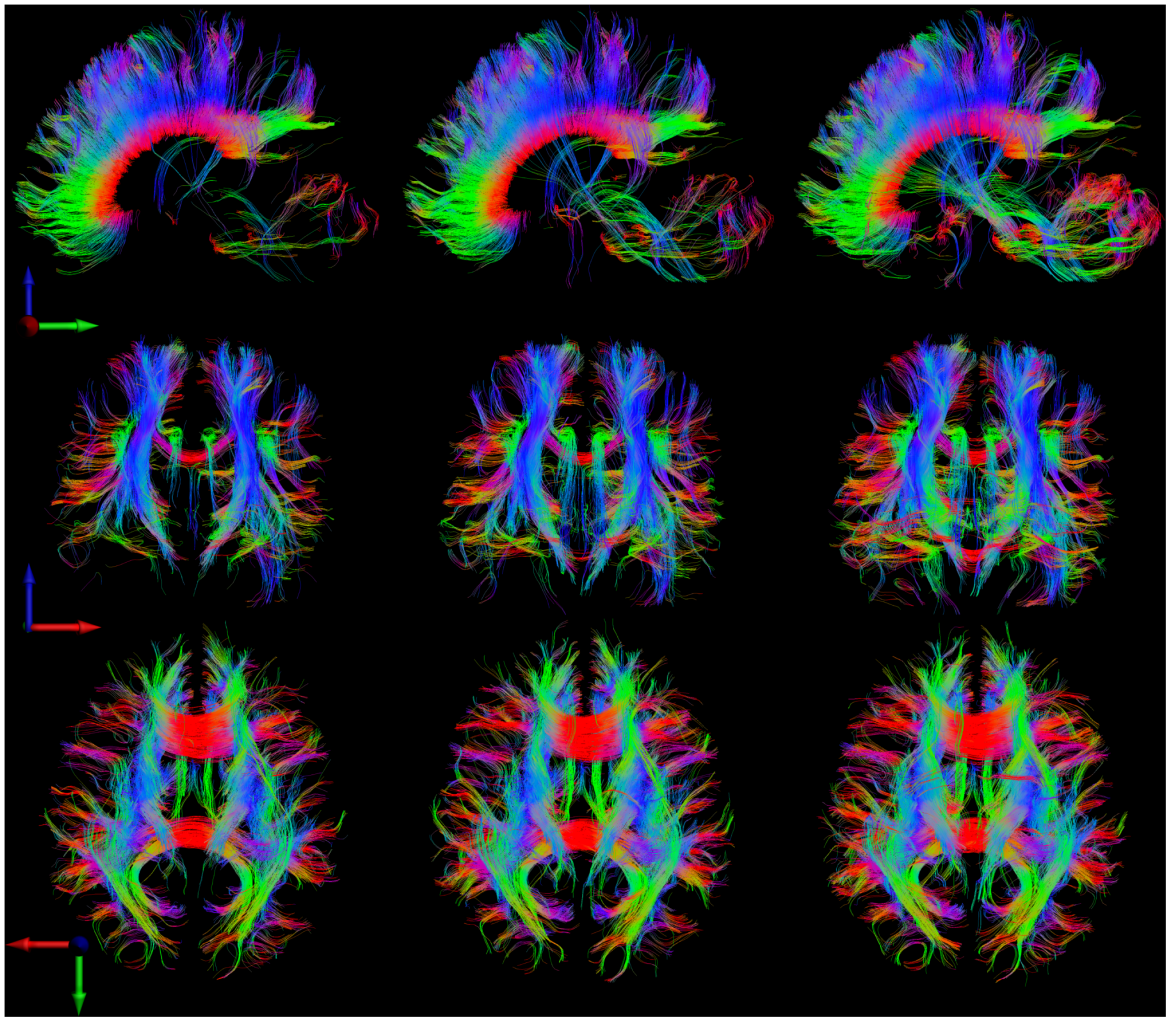
Fiber tracts through central slices: (x = 124) in sagittal view (first row), (y = 125) in coronal view (second row) and (z = 19) in transversal view (third row). The first column shows data from one measurement (first out of three averages), the second column from three averages and the third column from one measurement but after regularization. In certain regions, such as the center of the brain, the regularization algorithm reveals structures that can be observed in data from three averages but not in data from one average.

Consistent with the phantom results, for subject #1 the tractographic evaluation for in the vivo diffusion data revealed an increase in ML, TC, V, and VC as a function of number of averages (rs-EPI_1_<rs-EPI_2_<rs-EPI_3_) for the entire brain (see figure 5). For subject #2 we observed an increase in ML, TC, V, and VC given in that rs-EPI_1_<rs-EPI_2_ but no further improvement for rs-EPI_3_. Overall the regularized data (rs-EPI_1,reg_) showed the highest values for the evaluated parameters. Visually, an increase in fiber length and fiber density can be observed when comparing fiber tracts from one average with fiber tracts from three averages and with fiber tracts from the regularized data, respectively (see figure 6).

As expected, the ICA-EMB algorithm decomposed the images from 12 diffusion directions into six independent components containing information about tissue and diffusion, and six components representing noise (see figure 1). No additional component was observed confirming that the geometrical distortion correction was successful. The computing time for the ICA decomposition was about 70 minutes for all 38 slices (Intel® Core™ i7-2600 CPU, 3.4 GHz, 8 GB RAM).

**Figure 7:**
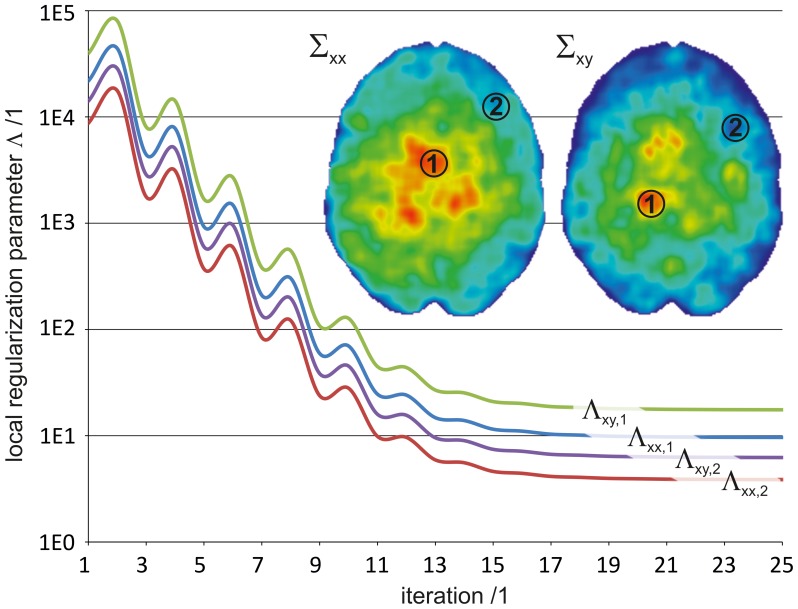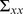 and 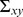.show the standard deviation of the noise for two of six noise tensor elements 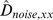 and 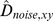 that serve as input for Eq. [13]. The spatially varying regularization parameter was updated at each iteration step. This is shown for two regions of interest (ROI 1, ROI 2) for the regularization tensor elements 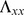 and 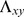.

**Figure 8:**
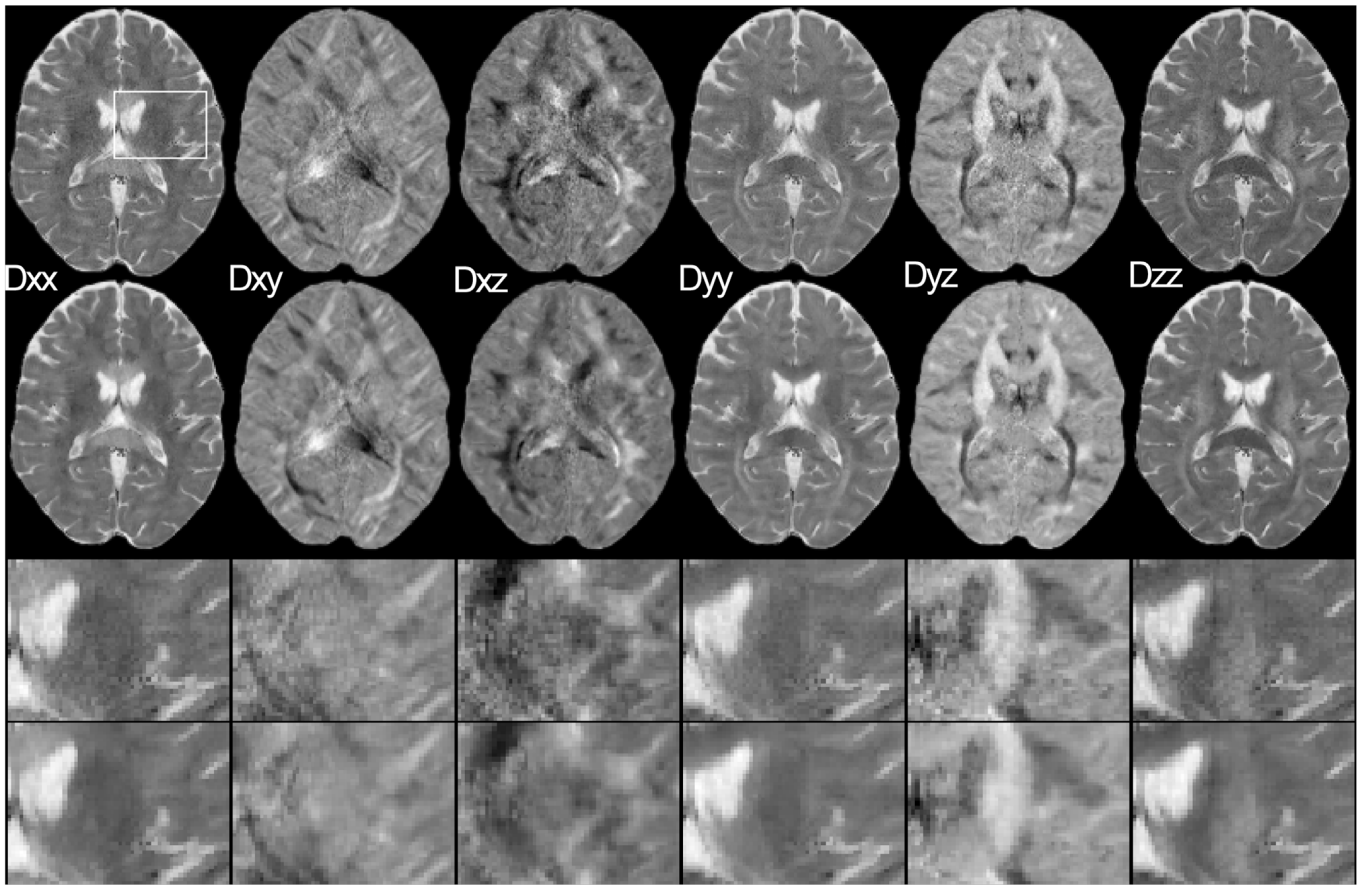
Diffusion tensor elements 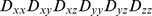 for one average (first row) and for one average regularized (second row). Rows three and four show a magnified view from the region marked in the first image.

**Figure 9:**
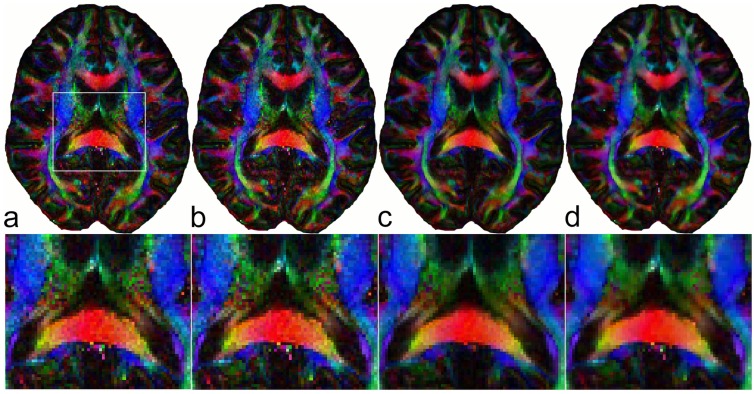
FA map from data obtained using one measurement (first out of three averages) (a), FA map from the denoised set of DWI data (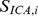) (b) showing only marginal differences compared with (a), FA map from data obtained using three averages (c), FA map from data obtained using one measurement (first out of three averages) with regularization (first row) (d). The second row shows magnified views from the region marked in the first image.

The noise, extracted from the DWI data by means of ICA, was projected into the tensor subspace. Figure 7 shows the standard deviation of the noise for the elements 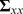 and 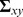. Due to the spatial inhomogeneity of the noise, modulated by physiology, g-factor in parallel imaging and normalization procedures, the standard deviation of the noise is higher in central regions of the brain compared to peripheral regions additionally highlighting the necessity of a spatially varying regularization. The standard deviation of the noise tensor served as a weighting factor for evaluating a spatially varying regularization tensor 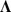. In every iteration step 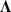 was updated according to Eq. [13]. Figure 7 exemplarily shows the evolution of the regularization parameter for two different regions, resulting in a higher regularization parameter for ROI 1 and a lower regularization parameter for ROI 2. The denoising properties of the regularization process are demonstrated in figure 8 for the individual diffusion tensor elements 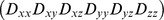 for the un-regularized data from one average and for the regularized data. In figure 9, FA maps are displayed for data from one average (a), one average with denoised DWI data (b), three averages (c) and one average with regularization (d).

## Discussion

In recent years, the increase in spatial resolution in diffusion-weighted imaging has gained attention in the scientific community since the usefulness of tractography has been demonstrated for many applications from psychological sciences to presurgical mapping. While technical developments enhancing the spatial resolution, such as ultra-high-field scanners or strong gradient systems, are limited to a few sites, developments in MR pulse sequence design can easily be adopted on clinical scanners. rs-EPI makes it possible to acquire high-resolution DWI data, but at the cost of scan time and SNR. To make this technique more applicable in a clinical setup, a method for scan time reduction has been implemented recently by omitting readout segments on one side of k-space and using Partial-Fourier reconstruction [56]. Another approach to increase the spatial resolution entitles ZOOPPA is based on scanning only a restricted field of view in combination with partially parallel acquisition [57]. In this paper, a complementary strategy has been presented, in which a regularization of the entire diffusion tensor is used to overcome the limitations of poor SNR at short scan times.

To test the performance of our regularization algorithm, we constructed a software phantom with fibers of specified lengths and known directions serving as a gold standard. For in vivo conditions the creation of a gold standard is only possible to a limited extent. Even though extended scan time due to the sampling of more averages results in more reliable DTI data, long scan times imply motion artifacts inherently limiting the data quality. Specifically our data show that the improvement when measuring 48 min is moderate compared to measurements obtained within 32 min (subject #1) or even slightly worse (subject #2). Hence, a gold standard cannot be created by prolonging the scan time ad infinitum. Despite these difficulties in comparing the tractography from the regularized data with a gold standard, a trend of increasing fiber density can be observed in the data from three averages suggesting that tractography results from the regularization process are plausible with respect to the anatomical structures underlying the diffusion measurements. It has to be noted, that of course data measured in 48 minutes contain more reliable information than measurements obtained in 16 minutes making tracking more robust to tracking errors. To what extent the regularization algorithm influences tracking errors positively or negatively can only be answered by comparison with the anatomy, a topic which remains under investigation for all tractographic algorithms. It also has to be mentioned, that anisotropic voxel are not ideal for fiber-tracking. For the sake of full brain coverage anisotropic voxel must be accepted to obtain data in a feasible time and are often used in clinical practice.

However, in this work we have shown for a software phantom and for high resolution in vivo data that our approach of regularizing the entire diffusion tensor is able to improve DTI-based tractography. For both, the phantom and in vivo data sets the mean fiber length is higher for the regularized data compared to the un-regularized data. This confirms that discontinuities in the main diffusion tensor direction, which account for stopping a fiber tract along its path, are corrected successfully. Related parameters such as Mean Length, Track Count, Voxel Count and Volume also show higher values for the regularized data compared to the un-regularized case. Specifically, for in vivo data these findings suggest that tractography results evaluated from regularized diffusion tensor data based on one measurement (first out of three averages) produce results more comparable to tractography results obtained from measurements with prolonged scan time. This significant 3-fold reduction of scan time makes rs-EPI DWI more applicable in a clinical context.

Our proposed method has two novel features. Firstly, the regularization parameter is evaluated automatically by being updated at every iteration step of the optimization algorithm, making the method user independent. The choice of the regularization parameter is crucial in denoising by means of regularization since it determines the trade-off between denoising and smoothing. In most regularization algorithms this value is chosen more or less arbitrarily, validated by visual inspection and is therefore always a subjective choice. In general, a prerequisite for the adoption of the regularization parameter is that the noise level for the data to be regularized has to be determined, which was carried out by means of ICA in this work. We directly calculated the noise distribution from the DWI data and thus eliminating the need of additional scans. Secondly, the regularization parameter varies spatially accounting for variations in SNR due to the spatial dependence of the coil sensitivity. Given the fact that the homogeneity of the coil sensitivity decreases with an increase of the number of coil elements this effect should not be neglected [40]. Specifically, when using head coils with 32 elements or more a constant regularization parameter will lead to over-regularization in cortical regions and to under-regularization in deep brain regions. Since spatially varying noise is not a central issue in most image processing applications this problem is often neglected. However, few methods exist to address this problem by estimating the noise from the data to be regularized [58]. Estimating the noise from an image to be regularized is not a trivial task and remains an approximation. Due to the multivariate nature of the DWI data, we could extract the noise by means of ICA and provide the basis for spatially varying regularization of the diffusion tensor after projecting the extracted noise into the tensor subspace. The distribution of the noise in the tensor space was evaluated using a sliding window including 5×5 pixels. Although the window size is not a crucial parameter with regard to the regularization outcome, it should be ensured that the window captures sufficient data points for estimating a reliable statistical distribution of the noise.

In the last decade, a multitude of algorithms have been proposed to solve the BSS problem using ICA. An overview can be found elsewhere [43]. Although the theoretical basis is different for many algorithms, the results are comparable providing components where the mutual statistical independence is maximized. We applied three different algorithms (ICA-EMB [53], FastICA [59], IM-ICA [41]) to DWI data and all of them separated tissue- and diffusion-related components from noise components sufficiently. The choice of using ICA-EMB in this work is motivated by the fact that the evaluated ICs are sorted so that the first six components represent tissue and diffusion related information. This is not a priori the case for all ICA algorithms since a permutation operator can always be applied to the basic ICA model, providing the same results in a different order. If the independent components are not sorted, the diffusion-related components or the noise-related components have to be identified manually in order to separate the tissue and diffusion signal from the noise signal. The number of six tissue- and diffusion-related components decomposed by ICA is independent from the number of diffusion sensitizing gradient directions. This can be explained by the fact that a symmetric tensor has six independent tensor elements and has been shown stable for a different number of gradient directions in our experiments.

It has to be noted that different ICA algorithms strongly vary in their computational effort and convergence speed. If processing speed is a crucial issue, the performance of different ICA algorithms should be considered. In case of geometrical distortions or motion artifacts additional independent components occur beside the parenchyma, diffusion components and noise components. Hence it is crucial to correct for these artifacts prior to performing ICA.

It has to be acknowledged that our method is only applicable to DWI measurements with more than six diffusion directions. Considering that ICA decomposes the DWI dataset into six components containing parenchyma and diffusion information and some components containing noise, it is obvious that more than six diffusion directions are required. Although this can be seen as a limitation of our method, most modern diffusion sequences allow the acquisition of 12 or more directions of the diffusion sensitizing gradient.

After the separation of tissue- and diffusion-related information from noise by means of ICA, only the components containing diffusion information were transformed back resulting in a denoised set of DWI data (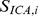). Using only the denoised DWI data for diffusion tensor imaging led approximately to the same result as that obtained from the original noisy DWI data (see figure 9 a and b). This interesting observation shows that denoising DWI data alone is not a successful strategy for improving diffusion tensor based parameters and regularization of the diffusion tensor itself is crucial. Considering Eq. [6] for solving the inverse problem to evaluate the diffusion tensor, the multiplication 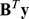 sums the pixel values from the 12 dimensional DWI space in order to perform a projection into the six dimensional tensor subspace. This summation has a denoising effect and explains the limited impact of using denoised DWI data for evaluating the diffusion tensor.

From a physical point of view the diffusion tensor is positive definite, meaning that the eigenvalues of the diffusion tensor must be positive. It must be guaranteed that this physical restriction is not violated after the regularization process and can be achieved by replacing the negative eigenvalues with zero [60]. This method has been shown to provide satisfying results compared with other methods [61].

Currently, our regularization approach is applied separately to each slice. Although a three-dimensional regularization seems to be the natural choice for improving tractography in a three-dimensional space, the realization is not trivial and some limitations complicate the implementation. A 3D regularization would enlarge the optimization problem by an additional dimension, solving the problem in a 240×240×6×38 space, which would result in a dramatic increase in computational effort. Furthermore the influence of the different resolutions for the in-plane and slice directions would have to be considered. With these points in mind, the feasibility of extending our algorithm to 3D is currently being investigated.

## Conclusions

In this work we introduced a novel regularization approach that is applied to the diffusion tensor from high-resolution readout-segmented DWI data. Using independent component analysis, noise information was extracted from DWI data and included in the regularization algorithm. Firstly, this allows an automatic evaluation of the regularization parameter, making our method user independent and secondly, it allows a spatially dependent regularization, accounting for inhomogeneities in SNR due to variances in coil sensitivity. Tractography and quantitative parameters from in vivo data showed that regularizing the diffusion tensor from DWI data measured in 16 minutes produces results that are comparable with DWI measurements from three averages obtained in 48 minutes. This significant reduction in scan time may advance the applicability of high resolution DTI and tractography in a clinical workflow.
